# *Andrographis paniculata* improves glucose regulation by enhancing insulin sensitivity and upregulating GLUT 4 expression in Wistar rats

**DOI:** 10.3389/fnut.2024.1416641

**Published:** 2024-10-31

**Authors:** W. A. Saka, O. S. Oyekunle, T. M. Akhigbe, O. O. Oladipo, M. B. Ajayi, A. T. Adekola, A. I. Omole, R. E. Akhigbe

**Affiliations:** ^1^Department of Physiology, Ladoke Akintola University of Technology, Ogbomoso, Nigeria; ^2^Department of Agronomy, College of Agricultural Sciences, Osun State University, Oshogbo, Nigeria; ^3^Reproductive Biology and Toxicology Research Laboratory, Oasis of Grace Hospital, Osogbo, Nigeria

**Keywords:** *Andrographis paniculata (Burm.f.) Nees [Acanthaceae]*, insulin resistance, diabetes, cardiometabolic disorder, glucose transport

## Abstract

**Context:**

Although the hypoglycaemic effect of *Andrographis paniculata (Burm.f.) Nees [Acanthaceae]* has been documented, reports on its effect in an apparently healthy state are limited.

**Objective:**

This study investigated whether or not *A. paniculata* exerts hypoglycaemic effect in a non-diabetic state. It also explored the impact of *A. paniculata* on glycolytic enzymes and GLUT 4 protein expression, as a possible mode of action.

**Methods:**

Twenty male Wistar rats were randomly assigned into two groups (*n* = 10 rats/group). The control rats were vehicle-treated (0.5 ml of distilled water), while the *A. paniculata*-treated rats had 500 mg/kg of *A. paniculata per os* once daily for 35 days.

**Results:**

*A. paniculata* treatment led to improved insulin sensitivity evidenced by increased HOMA-β (88.08 ± 2.13 vs. 120.80 ± 1.52, *p* < 0.0001), HOMA-S (283.60 ± 8.82 vs. 300.50 ± 9.30, *p* = 0.0189), and reduced TyG index (4.22 ± 0.04 vs. 3.95 ± 0.07, *p* < 0.0002) and HOMA-IR (0.32 ± 0.01 vs. 0.25 ± 0.01, *p* < 0.0001) when compared with the control. It also improved glucose regulation as depicted by reduced fasting blood glucose (3.77 ± 0.10 vs. 3.24 ± 0.11, *p* < 0.0001) and glycated hemoglobin (HbA1c; 7.69 ± 1.15 vs. 5.95 ± 0.82, *p* = 0.0245), and atherogenic dyslipidaemia, including AIP (−0.12 ± 0.03 vs. −0.26 ± 0.03, *p* < 0.0001) and CRI-I (2.70 ± 0.29 vs. 1.84 ± 0.27, *p* < 0.0001). These findings were accompanied by enhanced hepatic and muscular redox state, increased activities of glycolytic enzymes, upregulated GLUT 4 (0.80 ± 0.27 vs. 6.20 ± 0.84, *p* < 0.0001), and increased circulating nitric oxide (5.45 ± 0.24 vs. 6.79 ± 0.33, *p* = 0.0002).

**Conclusion:**

*A. paniculata* exerts positive effect on glucose metabolism and utilization by improving insulin sensitivity and upregulating the activities of glycolytic enzymes and GLUT 4 protein expression. This implies that *A. paniculata* may be beneficial in preventing insulin resistance and incident diabetes. Nonetheless, it should be used with caution to prevent hypoglycaemia in a non-diabetic state.

## Introduction

Glucose homeostasis is essential in the maintenance of life. Impaired glucose homeostasis results in insulin resistance (IR) that is the hallmark of glucose intolerance/pre-diabetic state and type II diabetes mellitus (T2DM) (1, 2). IR is increasingly becoming a major public health challenge (3) not just in the developed countries but also in the developing and under-developed countries (4, 5). Development of IR is coupled with reduced glucose uptake in insulin-sensitive tissues, especially hepatic and skeletal muscle. The transition of IR to T2DM involves cascades of inflammatory responses and oxidative stress-mediated signaling (6). This is accompanied by reduced level of circulatory nitric oxide (NO) due to the decline in its synthesis by uncoupling endothelial NO synthase (eNOS) via reactive oxygen species (ROS)-induced oxidation and depletion of tetrahydrobiopterin (BH_4_) (4, 5, 7).

Insulin is the most potent physiological agent that maintains glucose regulation (8). This is primarily achieved by upregulating the expression of glucose transporter 4 (GLUT 4) at the plasma membrane (9, 10) by the translocation of GLUT4 (11, 12). Hence, the rate of glucose transport is influenced by GLUT 4 protein expression, and disruption of GLUT4 expression has been linked with impaired glucose uptake, IR and T2DM (13, 14).

Also, insulin upregulates the expression of genes that encode glycolytic enzymes (15). Ingested carbohydrates are broken down by amylase, which initiate glucose metabolism. Glucose is esterified into glucose-6-phosphate (G-6-P) by hexokinase (16). G-6-P is isomerized into fructose-6-phosphate (F-6-P), which is further converted into fructose-1,6-biphosphate (F-1,6-BP) by phosphofructokinase-1 (15). This is the rate-limiting step in the glycolytic pathway. F-1,6-BP undergoes series of oxidative breakdown to yield phosphoenol pyruvate (PEP), which is broken down into pyruvate by pyruvate kinase (17). Alternatively, G-6-P may be converted into 6-phosphogluconolactone, which enters the pentose phosphate pathway, an alternative route of glucose oxidation, to also generate energy in the form of adenosine triphosphate (ATP) under the action of glucose-6-phosphate dehydrogenase (G-6-PD) (15).

In recent times, a number of herbal nutraceuticals have been revealed to protect against IR and incident T2DM (18), however, few have been well studied. One of such nutraceutical is *Andrographis paniculata (Burm.f.) Nees [Acanthaceae]*. *A. paniculata*, a botanical with antioxidant and anti-inflammatory properties (19), has been shown to possess anti-hyperglycemic activities in diabetic animal model by protecting pancreatic β-cells (20, 21) and mitigating oxidative and inflammatory markers (22, 23). In another laboratory study, Chen et al. (24) demonstrated that *A. paniculata* attenuated high fat diet-induced IR by ameliorating inflammation-driven impairment of insulin resistance. The activities of *A. paniculata* have been ascribed to its high diterpenoid, polyphenolic and flavonoid contents (19, 23). Andrographolide, a diterpenoid, is the major metabolite of *A. paniculata* (19). Apart from being used as a supplement or herbal remedy in folklore medicine, the root, seed, and leaves are also macerated and taken as aperitive; hence apparently healthy individuals also consume this botanical. Despite the available pieces of evidence in the literature on the hypoglycemic effect of *A. paniculata*, studies reporting the impact of this nutraceutical in an apparently healthy state are limited. Also, the role of GLUT 4, and phosphofrucokinase and G-6-PD, the rate limiting enzymes in glycolytic and pentose phosphate pathways respectively, have not been elucidated.

A lot of times, researchers consider the importance of a botanical as a drug and test it in a model of human pathology such as diabetes. However, these botanical could be used as supplements to prevent the incident of human pathologies. Hence, it is important to test the effect of such botanicals like *A. paniculata* in normal state. Therefore, this study was designed to investigate whether or not *A. paniculata* exerts hypoglycaemic effect in a non-diabetic state, and to explore the role of glycolytic enzymes, including phosphofrucokinase and G-6-PD, and GLUT 4 in *A. paniculata*-induced glucose regulation.

## Materials and methods

### Animals

This study was conducted according to the National Institutes of Health Guide for the Care and Use of Laboratory Animals, and was approved by the Ethical committee of the Faculty of Basic Medical Sciences, Ladoke Akintola University of Technology, Ogbomoso, Oyo State, Nigeria (FBMS/AEC/P/074/22). Conscious efforts were made to minimize the number of rats used and their suffering. Twenty eight weeks old male Wistar rats of comparable weight were used for the experiment. Animals were procured from the animal house of the Department of Physiology, Ladoke Akintola University of Technology (LAUTECH), Ogbomoso, Nigeria. The animals were housed in ventilated plastic cages (5 rats/cage) and acclimatized for 2 weeks before the commencement of the experiment at room temperature. Rats were allowed unrestricted access to pelletized animal feed and water. The rats were randomly assigned to vehicle-treated control (Control) and *A. paniculata*-treated (Treated) groups (*n* = 10 rats/group).

### Treatment

The control group received distilled water (vehicle-treated control; *per os*), while the treated group received 500 mg/kg of *A. paniculata per os*, once daily for 35 days. The dose and route of administration was as previously established and reported (19). *A. paniculata* leaf powder was used to mimic its popular mode of use in humans as previously reported (19, 25). The fresh leaves of *A. paniculata* were commercially procured and authenticated by Dr. Mrs. Ogundola, a botanist at LAUTECH. The leaves were air-dried and pulverished using an electric blender. The obtained powder was dissolved in distilled water (2 mg of *A. paniculata* powder in 1 ml of distilled water) for use (19). The dose of *A. paniculata* used in the present study is based on the findings of our pilot study and also similar with some previously reported studies (19, 25, 26).

### Phytochemical analysis of *A. paniculata*

The qualitative analysis of *A. paniculata* carried out as earlier reported for phytochemical analysis (27). Briefly, for alkaloids determination, 1 ml of aqueous *A. paniculata* leaf extract was stirred with 5 ml of 1% (v/v) aqueous HCl on a steam bath and filtered while hot. Distilled water was added to the residue and then 1 ml of the filtrate was treated with a few drops of Mayer’s reagent, Wagner’s reagent and Dragendoff’s reagent. The presence of alkaloids was confirmed by the formation of a yellow color with Mayer’s reagent, reddish-brown precipitate with Wagner’s reagent and red precipitate with Dragendoff’s reagent.

For determination of the presence of tannins, 1 ml of aqueous *A. paniculata* leaf extract was boiled in 20 ml of distilled water in a test tube and then filtered. Three drops of 0.1% ferric chloride was added to the filtrate. The presence of tannins was confirmed by the formation of a green color.

For terpenoids, 5 ml of aqueous *A. paniculata* leaf extract was mixed with 2 ml of chloroform. Three milliliter of concentrated H_2_SO_4_ was then carefully added to form a layer. The presence of terpenoids was confirmed by reddish-brown discoloration of the interface.

For flavonoids, 1 ml of 10% (w/v) NaOH was added to 3 ml of aqueous *A. paniculata* leaf extract. The presence of flavonoids was confirmed by the formation of a yellow color.

For saponins, 5 ml of aqueous *A. paniculata* leaf extract was boiled in 20 ml of distilled water in a water bath and filtered. Precisely, 10 ml of the filtrate was mixed with 5 ml of distilled water and shaken vigorously to obtain a stable persistent froth. The resulting froth was then mixed with three drops of olive oil and shaken vigorously. The formation of emulsion indicated the presence of saponins.

For glycosides, 5 ml of the aqueous *A. paniculata* leaf extract was added to 2 ml of glacial acetic acid containing one drop of ferric chloride solution. This was then underplayed with 1 ml of concentrated sulphuric acid. The formation of a violet-green ring below the brown ring confirmed the presence of glycosides.

For steroids, 2 ml of acetic anhydride was added to 2 ml of the aqueous *A. paniculata* leaf extract followed by the addition of 2 ml of concentrated H_2_SO_4_. The presence of steroids was confirmed by a colour change from violet to blue or green.

### Glucose homeostasis

Oral glucose tolerance test (OGTT) was conducted 24 h before the termination of the experiment. Blood sample was obtained from the tail of each rat before glucose load (0 min) for glucose assay. Afterwards, rats were orally loaded with glucose (2 g/kg b.w) and blood samples were obtained at 30, 60, 90, and 120 min for blood glucose determination. At the end of the experiment, blood sample was obtained from 12 h overnight-fasted rats for fasting blood glucose. Blood glucose concentrations were determined using an ACCUCHEK glucometer (Roche Diabetes Care Inc., Basel, Switzerland). Glucose tolerance was expressed as a function of the area under the OGTT curve (AUC).

### Sample collection

At the end of the study, overnight-fasted rats were weighed and the body weight change was determined as the difference between the final body weight (body weight at the end of the study) and the initial body weight (body weight at the start of the study). Rats were euthanized with ketamine (40 mg/kg) and xylazine (4 mg/kg), which were administered intraperitoneally (28). Blood samples were collected via the retro-orbital vein into appropriate sample bottles. Blood samples were centrifuged at 3,000 rpm for 5 min to obtain the serum, which was stored frozen until needed for biochemical assay. The liver and gastrocnemius muscle were excised, cleared of adhering tissues, blotted and weighed. The hepatic and half of the gastrocnemius tissues were homogenized in a glass homogenizer, centrifuged at 10,000 rpm for 10 min at 4°C to obtain the supernatant, which was stored frozen until needed for biochemical assay. The other half of the gastrocnemius tissue was used for GLUT 4 assay using histoimmunochemistry.

### Biochemical assay

Plasma insulin level was determined using ELISA kit from DiaSorin (Saluggia, Italy). Glycated haemoglobin (HBA1c) was estimated by ion exchange resin method as earlier reported (29). Insulin sensitivity was determined using homeostasis model assessment (HOMA), qualitative insulin check index (QUICKI), and triglyceride-glucose ratio (TyG index, an index of insulin resistance) (30, 31).


$$\begin{array}{l} \text{HOMA}-\beta \;\left(\text{pancreatic}\;\beta \;\text{cell function}\right)=\\ 20\times \text{fasting blood insulin level}\;\left(\mu U/\text{ml}\right)/\\ \text{fasting blood glucose}\;\left(\text{mmol}/L\right)-3.5 \end{array}$$

$$\text{HOMA}-S\;\left(\text{insulin sensitivity}\right)=\left[1/\text{HOMA}-IR\right]\times 100\%$$

$$\begin{array}{l} \text{HOMA}-IR\;\left(\text{insulin resistance}\right)=\\ \text{Fasting glucose concentration}\;\left(\text{mmol}/L\right)\times \\ \text{fasting blood insulin concentration}\;\left(\mu U/\text{ml}\right)/22.5 \end{array}$$

$$\begin{array}{l} \text{QUICKI}=(1/[\log\;\left(\text{fasting insulin level}\right)\\ \;+\log\;\left(\text{fasting glucose level}\right)]) \end{array}$$

$$\text{TyGindexLn}\left[\text{Fastingtriglyceridemg}/\text{dlfastingglucosemg}/\text{dl}\right]/2$$

Serum lipids (total cholesterol, TC, triglyceride, TG, low density lipoprotein, LDL-C, and high density lipoprotein, HDL-C) were determined by standard colorimetric methods using laboratory reagents (Randox Laboratory Ltd., Antrim, UK) following the manufacturer’s guideline. Atherogenic index of plasma (AIP), Castelli’s risk index-I (CRI-I), and Castelli’s risk index-II (CRI-II) were calculated as atherogenic indices using Log TG/HDL-C, TC/HDL-C, LDL-C/HDL-C, respectively (30).

Serum amylase and hepatic hexokinase, phosphofructokinase, pyruvate kinase and G6PD activities were assayed as carbohydrate-metabolizing and glycolytic enzymes by colorimetric method per the manufacturer’s guideline (Randox Laboratory Ltd., UK).

Serum total protein (Randox Laboratory Ltd., UK), albumin (Agappe, India), and bilirubin (Randox Laboratory Ltd., UK), as well as hepatic activities of aspartate transaminase (AST; Agappe, India), alanine transaminase (ALT; Randox Laboratory Ltd., UK), alanine phosphatase (ALP; Randox Laboratory Ltd., UK), and γ-glutamyl transferase (Pointe Scientific Inc., USA) were used as indices of hepatic function and spectrophotometrically determined per manufacturers’ instructions as previously reported (32).

The activities of lactate dehydrogenase (LDH; Randox Laboratory Ltd., UK), creatinine kinase (CK; Agappe Diagnostics, Switzerland), and aspartate transaminase (AST; Agappe, India) in the gastrocnemius muscle were determined as muscle injury biomarker, and assayed by colorimetric method per the manufacturer’s guideline as previously reported (33).

Hepatic and muscular levels of malondialdehyde (MDA), marker of oxidative stress, were determined by standard enzymatic-colorimetric methods (Fortress Diagnostic, Antrim, UK). Hepatic and muscular levels of reduced glutathione (GSH) and superoxide dismutase (SOD) activities, antioxidant markers, were assayed using colorimetric methods as previously documented (27, 34).

Hepatic and muscular concentrations of tumour necrosis factor-α (TNF-α) and interleukin-1β (IL-1β), markers of inflammation, were determined using standard ELISA kit (Elabscience Biotechnology Co., Ltd., USA) per manufacturer’s guideline.

Serum nitric oxide (NO) was determined by non-enzymatic colorimetric assay (Oxford Biomedical Research Inc., Rochester Hills, USA).

### Immunohistochemical analysis of GLUT 4

Immunohistochemical analysis and scoring were done as previously reported (35). Formalin-fixed and paraffin-embedded gastrocnemius muscular tissues were sectioned at about 4 μm for immunohistochemistry. The procedures were performed using Thermo Fischer Kit (Thermo Fischer Scientific Inc., USA) and anti-mouse GLUT4 monoclonal for GLUT4 expression (Thermo Fischer Scientific Inc., USA). After de-paraffinization and rehydration of the sections, the antigen was retrieved using pre-heated citrate buffer and allowed to cool for 30 min. The slides were cleaned with Kim wipes, section areas marked with a hydrophobic pen, and slides were then arranged in a humidified chamber. The slides were incubated for 10 min following blockade of endogenous peroxidase activity using hydrogen peroxide. The slides were rinsed once with phosphate buffer saline (PBS) and ultra V protein block was applied and incubated for 10 min. Then, the slides were rinsed twice with PBS, GLUT4 primary antibody was applied. The slides were incubated for 45 min and rinsed with PBS twice again, then the primary antibody amplifier (secondary antibody) was applied. Thereafter, the slides were incubated for 25 min, rinsed with PBS twice, and HRP polymer was added. This cycle was repeated, then sections were incubated for 5 min in diaminobenzidine (DAB) substrate, rinsed twice with PBS, counterstained with Haematoxylin and rinsed with distilled water. Blueing solution was applied to the sections and rinsed, dehydrated, cleared, and mounted for qualitative examination. For quantification, digital photomicrographs obtained were imported unto Image J Software (NIH, Bethesda, MD, USA) with specific plugins for analysis of the intensity of the staining and percentage positive cells for GLUT4. The results were expressed as fold change relative to the normal control group.

### Statistical analysis

Statistical analysis was conducted using Graph Pad Prism (version 5.0). Unpaired Student’s *T*-test was performed to test for significance. Data are expressed as mean ± SD. Statistical significant differences were accepted at *p* < 0.05.

## Results

### Phytochemical constituents of *A. paniculata*

Findings of the qualitative phytochemical analysis revealed that the aqueous leaf extract of *A. paniculata* contained low concentrations of saponins, glycosides, and phytosterols and high concentrations of terpenoids, alkaloids, tannins, and flavonoids (Table 1).

**Table 1:** Phytochemical constituents of *Andrographis paniculata*.

Phytochemical	Presence
Alkaloids	++
Tannin	++
Saponin	+
Terpenoids	++
Flavonoids	++
Glycoside	+
Phytosterol	+

−: not detected; +: present in low concentration; ++: present in moderate concentration.

### Effect of *A. paniculata* on body weight

There was no difference in the body weight gain of the *A. paniculata-*treated rats when compared to the control (Table 2). Although there was a fall in body weight gain in *A. paniculata-*treated rats when compared to the control, the difference was only marginal.

**Table 2:** Effect of *Andrographis paniculata* on body weight change.

	Control	Treated
Initial body weight (g)	166.70 ± 1.22	167.10 ± 1.30
Final body weight (g)	195.61 ± 2.10	195.40 ± 2.02
Body weight change (g)	28.90 ± 1.12	28.30 ± 1.09

Data are expressed as mean ± SD for 10 rats per group and analyzed by unpaired *T*-test.

### Effect of *A. paniculata* on glucose homeostasis and atherogenic lipid

**Figure 1:**
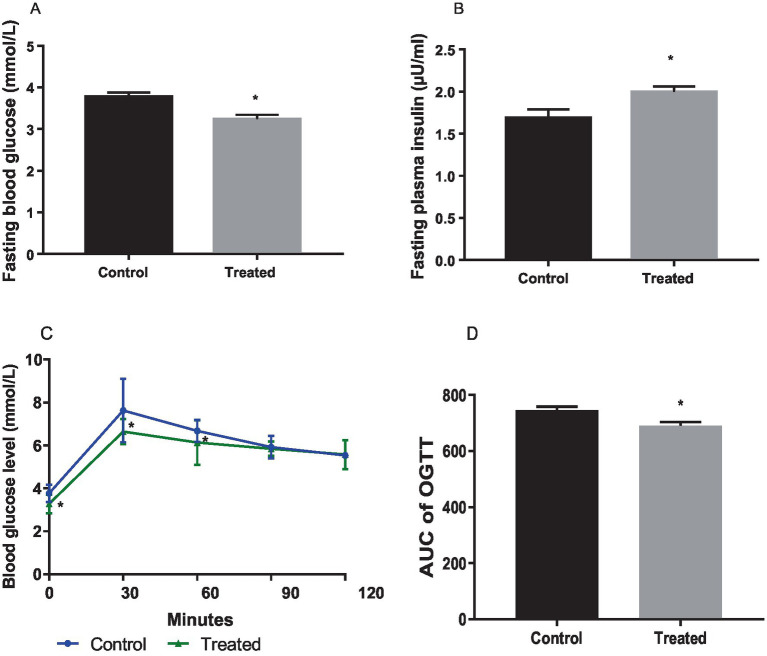
Effect of *Andrographis paniculata* on fasting blood glucose (A), fasting plasma insulin. (B), oral glucose tolerance test (OGGT, C), and area under the OGTT curve (AUC, D). Data are expressed as mean ± SD for 10 rats per group and analyzed by unpaired *T*-test. *p < 0.05 vs. control.

Terminal fasting blood glucose was significantly reduced in *A. paniculata-*treated rats (Figure 1A), while fasting insulin (Figure 1B) concentration was increased in *A. paniculata*-treated rats when compared with the vehicle-treated control. *A. paniculata* treatment led to a fall in 30 min and 1 h post-load glycaemia following oral glucose challenge when compared with the control group (Figure 1C). In addition, *A. paniculata* treatment caused a decrease in the area under the curve of OGTT when compared with the control (Figure 1D). There was also a rise in circulatory insulin 30 min following oral glucose challenge in the *A. paniculata*-treated rats when compared with the control.

**Figure 2:**
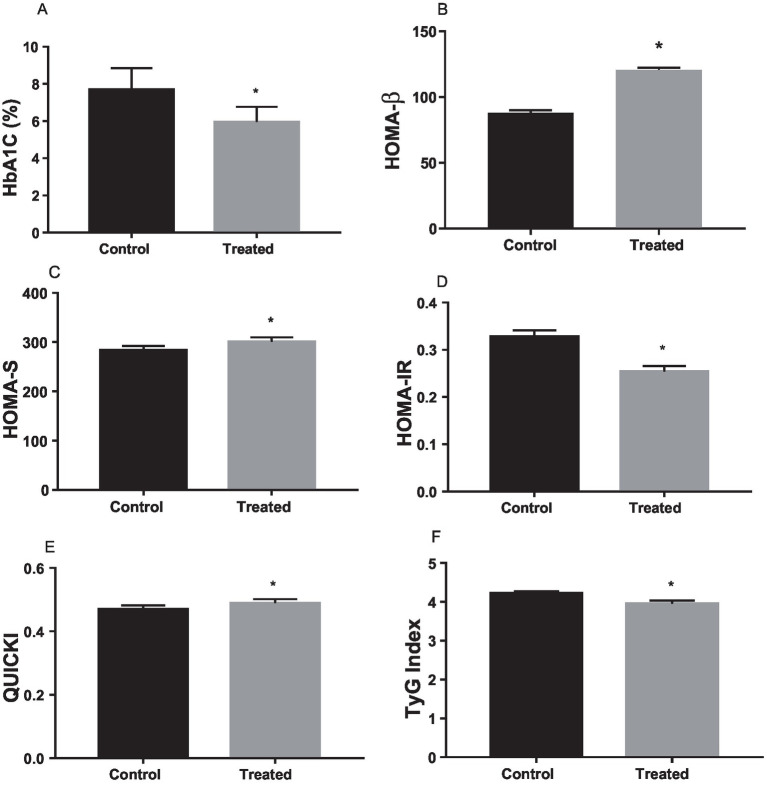
Effect of *Andrographis paniculata* on carbohydrate metabolizing and glycolytic enzymes such as serum amylase **(A)**, hexokinase **(B)**, phosphofructokinase **(C)**, pyruvate kinase **(D)**, and glucose 6 phosphate dehydrogenase (G6PD) activities **(E)** Data are expressed as mean ± SD for ten rats per group and analyzed by unpaired *T*-test *p < 0.05 vs control.

*A. paniculata* treatment significantly reduced glycated haemoglobin level when compared with the control (Figure 2A), which is indicative of good glycaemic control with *A. paniculata* treatment. Also, *A. paniculata* treatment led to significant increase in pancreatic β cell function evident by a rise in HOMA-β (Figure 2B). Furthermore, *A. paniculata* treatment significantly enhanced insulin sensitivity and reduced insulin resistance evident by a significant rise in HOMA-S (Figure 2C), a reduction in HOMA-IR (Figure 2D), an increase in QUICKI (Figure 2E), and a reduction in TyG (Figure 2F).

Treatment with *A. paniculata* significantly reduced circulating TC, TG, and LDL-C when compared with the vehicle-treated control group (Table 3). However, there was an increase in serum HDL-C in *A. paniculata*-treated rats when compared with the control rats (Table 3). Furthermore, there was a decrease in the indices of atherogenic dyslipidaemia (AIP and CRI-I) in the *A. paniculata*-treated rats when compared with the control rats. However, CRI-II was comparable in both the control and *A. paniculata*-treated groups (Table 3).

**Table 3:** Effect of *Andrographis paniculata* on serum lipids and atherogenic indices.

	Control	Treated
TC (mg/dl)	98.45 ± 2.92	81.75 ± 2.44^*^
TG (mg/dl)	60.80 ± 3.27	54.58 ± 3.34^*^
LDL-C (mg/dl)	39.05 ± 2.63	34.02 ± 3.71^*^
HDL-C (mg/dl)	36.49 ± 2.71	43.86 ± 4.68^*^
AIP	−0.12 ± 0.02	−0.26 ± 0.03^*^
CRI-I	2.70 ± 0.29	1.83 ± 0.27^*^
CRI-II	1.08 ± 0.32	0.70 ± 0.29

TC, total cholesterol; TG, triglyceride; HDL-C, high-density lipoprotein; AIP, atherogenic index of plasma; CRI-I, Castelli risk index I; CRI-II, Castelli risk index II. Data are expressed as mean ± SD for 10 rats per group and analyzed by unpaired *T*-test. *p < 0.05 vs. control.

### Effect of *A. paniculata* on glycolytic enzymes and muscular GLUT 4 expression

**Figure 3:**
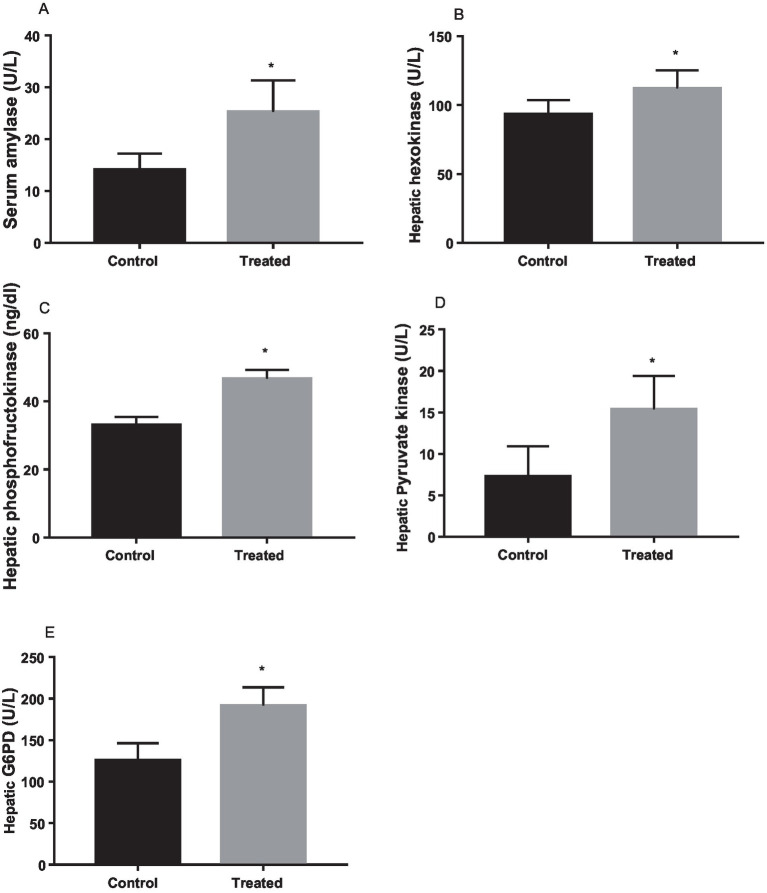
Effect of *Andrographis paniculata* on carbohydrate metabolizing and glycolytic enzymes such as serum amylase **(A)**, hexokinase **(B)**, phosphofructokinase **(C)**, pyruvate kinase **(D)**, and glucose 6 phosphate dehydrogenase (G6PD) activities **(E)**. Data are expressed as mean ± SD for ten rats per group and analyzed by unpaired *T*-test. *p < 0.05 vs control.

**Figure 4:**
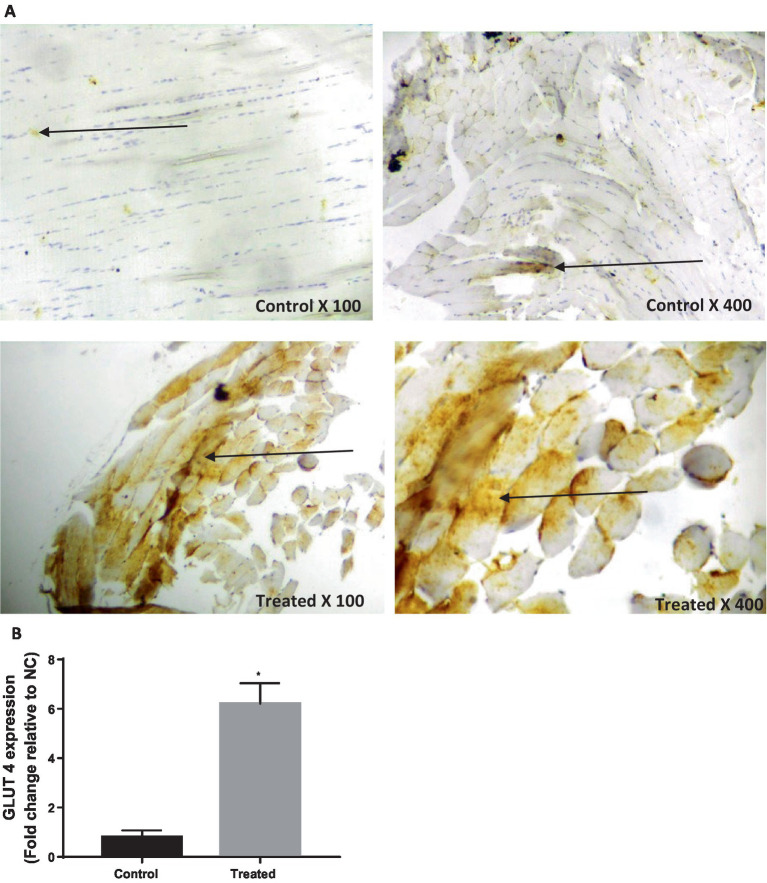
Effect of *Andrographis paniculata* on glucose transporter 4 (GLUT 4) expression (A) and numerical data representing GLUT 4 expression (B). Data are expressed as mean ± SD for 10 rats per group and analyzed by unpaired *T*-test. *p < 0.05 vs. control.

Treatment with *A. paniculata* caused an increase in the activities of serum amylase and hepatic hexokinase, phosphofructokinase, pyruvate kinase, and G-6-PD when compared to the vehicle-treated control (Figure 3). There was also an increase in the expression of GLUT 4 in the gastrocnemius muscle of the *A. paniculata*-treated rats when compared with the control (Figure 4).

### Effect of *A. paniculata* on muscle and liver function biomarkers

**Figure 5:**
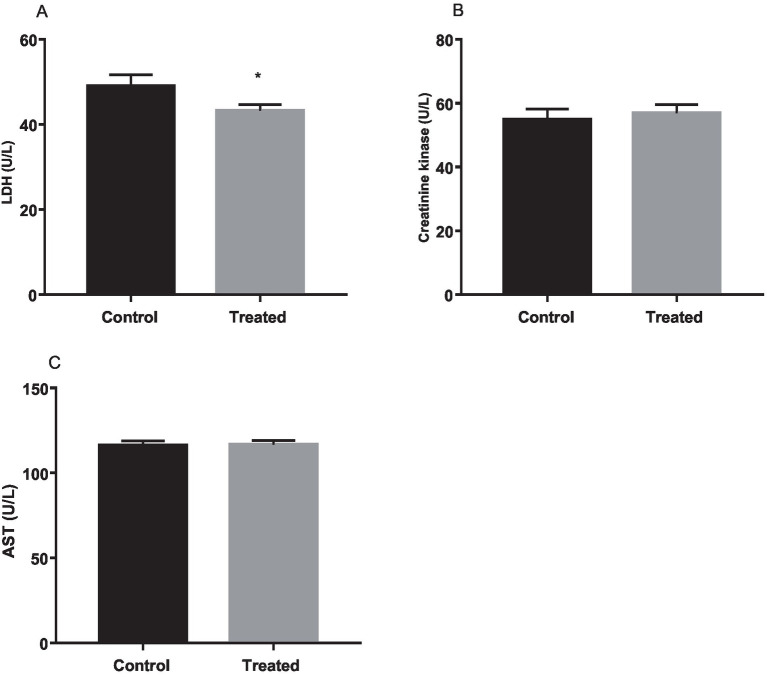
Effect of *Andrographis paniculata* on muscle injury markers; lactate dehydrogenase (LDH, A), creatinine kinase (B), and aspartate transaminase (AST, C). Data are expressed as mean ± SD for 10 rats per group and analyzed by unpaired *T*-test. *p < 0.05 vs. control.

Results from the present study revealed that treatment with *A. paniculata* led to a significant decrease in muscular activity of LDH when compared with the control. However, muscular activities of CK and AST were comparable between the control and *A. paniculata*-treated rats (Figure 5). Interestingly, serum levels of total protein, albumin, and bilirubin, as well as the hepatic activities of AST, ALT, ALP, and GGT where comparable with the vehicle-treated control (Table 4).

**Table 4:** Effect of *Andrographis paniculata* on hepatic function markers.

	Control	Treated
Total protein (g/dl)	5.46 ± 0.18	5.52 ± 0.15
Albumin (g/dl)	2.26 ± 0.86	1.98 ± 0.29
Bilirubin (mg/dl)	2.87 ± 0.21	2.96 ± 0.31
AST (U/L)	145.10 ± 21.83	132.20 ± 19.32^*^
ALT (U/L)	211.90 ± 19.53	207.60 ± 22.60
ALP (mg/dl)	47.29 ± 1.90	47.84 ± 1.26
GGT (U/L)	43.72 ± 4.13	35.55 ± 3.69*

AST, aspartate transaminase; ALT, alanine transaminase; ALP, alanine phosphatase; GGT, gamma glutamyl transferase. Data are expressed as mean ± SD for ten rats per group and analyzed by unpaired *T*-test. *p < 0.05 vs. control.

### Effect of *A. paniculata* on oxidative stress and pro-inflammatory biomarkers

**Figure 6:**
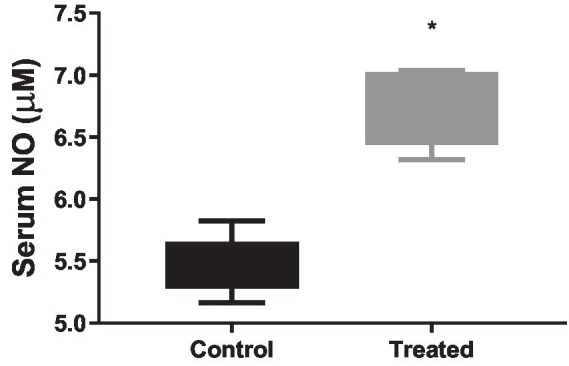
Effect of *Andrographis paniculata* on circulatory nitric oxide (NO). Data are expressed as mean ± SD for 10 rats per group and analyzed by unpaired *T*-test. *p < 0.05 vs. control.

*A. paniculata* treatment led to reduction in muscular and hepatic oxidative stress as evidenced by decline in muscular and hepatic MDA found in *A. paniculata*-treated rats when compared with the control animals (Table 5). In addition, there were increased levels of GSH and SOD activities in the muscular and hepatic tissues in *A. paniculata*-treated animals when compared with the control animals (Table 5). More so, treatment with *A. paniculata* significantly reduced muscular and hepatic levels of TNF-α and IL-1β when compared with the control rats (Table 6). Also, there was a rise in the circulating level of NO in *A. paniculata*-treated rats when compared with the control group (Figure 6).

**Table 5:** Effect of *Andrographis paniculata* on markers of oxidative stress.

	Control	Treated
Hepatic		
MDA (uM)	15.09 ± 0.77	11.95 ± 1.39^*^
GSH (mM)	0.42 ± 0.05	0.58 ± 0.06^*^
SOD (U/mg)	1.99 ± 0.12	2.19 ± 0.32^*^
Muscular		
MDA (uM)	8.00 ± 1.00	3.80 ± 0.83^*^
GSH (mM)	0.24 ± 0.02	0.38 ± 0.01^*^
SOD (U/mg)	0.58 ± 0.08	0.92 ± 0.06^*^

MDA, malondialdehyde; GSH, reduced glutathione; SOD, superoxide dismutase. Data are expressed as mean ± SD for 10 rats per group and analyzed by unpaired *T*-test. ^*^p < 0.05 vs. control.

**Table 6:** Effect of *Andrographis paniculata* on markers of inflammation.

	Control	Treated
Hepatic		
TNF-α (pg/mg)	16.13 ± 1.31	12.07 ± 1.17^*^
IL-1β (pg/mg)	15.44 ± 1.09	9.94 ± 0.96^*^
Muscular		
TNF-α (pg/mg)	13.73 ± 1.40	11.25 ± 1.80^*^
IL-1β (pg/mg)	12.64 ± 1.22	8.56 ± 1.25^*^

NO, nitric oxide; TNF-α, tumour necrosis factor-α; IL-6, interleukin-6. Data are expressed as mean ± SD for 10 rats per group and analyzed by unpaired *T*-test. ^*^p < 0.05 vs. control.

## Discussion

The current study demonstrates that *A. paniculata* improves glucose regulation and atherogenic lipids, which is coupled with upregulation of the activities of carbohydrate-metabolizing enzymes and GLUT 4 protein expression. IR is rapidly becoming a major public health challenge globally (3–5) with an increased risk of T2DM (1, 2). Individuals with IR have been reported to exhibit hyperglycaemia, low circulating NO, and reduced activity of carbohydrate-metabolizing enzymes and GLUT 4 protein expression (4), while *A. paniculata*, a herbal nutraceutical commonly used in folklore medicine for a myriad of pathological conditions, has been shown to exert anti-hyperglycaemic effect (21, 23, 36). Hence, our present finding that *A. paniculata* improves glucose regulation is not just in consonance with these earlier reports; it is an extension of the available data in the literature on the hypoglycaemic effect and the associated gluco-regulatory mechanisms of *A. paniculata* in a non-diabetic state.

The finding in this study that *A. paniculata* improves glucose regulation coupled with reduced 1-h post-load glycaemia in a non-diabetic state is worthy of note. This is because this observation may infer that although *A. paniculata* may exert anti-hyperglycaemic effect in a diabetic condition as previously reported, it also reduces the risk of developing IR and incident diabetes in a non-diabetic state. It has been reported that elevated 1-h post-load glycaemia is a valid predictor of pancreatic β-cell dysfunction, IR, and atherosclerotic cardiovascular diseases (CVD) (37). Another study has demonstrated that 1-h post-load glycaemia is a stronger independent indicator of pancreatic β-cell impairment and cardiometabolic disorders than 2-h post-load glycaemia (38). The area under the curve (AUC) indicates the amount of glucose in the circulation following a 75 g of glucose challenge (39). A higher AUC shows poor glucose clearance which indicates low insulin sensitivity and poor glucose regulation, while a lower AUC indicates good glucose clearance which suggests enhanced insulin sensitivity and improved glucose regulation. The present findings that *A. paniculata* reduced the AUC and circulating glucose level may be ascribed to its ability to enhance insulin sensitivity and optimize glucose regulation. *A. paniculata* did not only increased insulin sensitivity, it also increased the β cell function, thus increased insulin secretion. Thus, it is safe to infer that *A. paniculata* improved glucose regulation by upregulating β cell function and insulin secretion, and improving insulin sensitivity.

Furthermore, studies have revealed that atherogenic dyslipidaemia is a potent marker in identifying the risk of developing atherosclerotic CVD (40, 41). Thus, the finding that *A. paniculata* significantly reduced atherogenic dyslipidaemia, especially circulating HDL-C, AIP, and CR-I is remarkable. AIP, CR-I and CR-II are derivatives of lipid profile and are predictors of cardiovascular diseases. Since these indices are key predictors of coronary artery disease and cardiometabolic disorder (42), an increase in any or all of these variables is suggestive of increased risk of cardiometabolic disease. While CR-II considers the level of LDL and HDL, CR-I considers total cholesterol and HDL, hence both are better predictors of cardiometabolic and coronary artery disease than the traditional lipids (43). *A. paniculata*-induced improvement of dyslipidaemia was observed to be associated with unaltered body weight. This suggests that in an apparently healthy state, *A. paniculata* treatment may confer protection on cardiometabolic factors by improving atherogenic indices while sparing body weight change. Hence, when compared with the vehicle-treated control, the present findings that *A. paniculata* treatment enhanced insulin sensitivity and glucolipid regulation, which is accompanied by improvement of atherogenic dyslipidaemia demonstrates the potential protective effect of *A. paniculata* on pancreatic β-cell function, and incident IR and cardiometabolic disorders.

Reactive oxygen species (ROS) are generated during metabolic function (4). At low or moderate levels, they play physiological roles such as homeostatic function, signaling processes, and defence mechanisms (44); however, when they are generated in excess, they cause an imbalance in cellular redox state, leading to oxidative stress with attendant damage to the cellular lipid (lipid peroxidation), protein (protein denaturation) and DNA (DNA damage) (4). Oxidative stress is a key player in the pathogenesis of IR and cardiometabolic disorders (5, 45). Lipid peroxidation is the oxidative damage to the phospholipid layer of the cell membrane and cellular organelles, resulting in conformational changes and impairment in cell membrane and organelle function, which could result in cell death (28). This process generates MDA as an end product (4); hence, MDA is used as a biomarker of oxidative stress. Therefore, the finding in the present study that *A. paniculata* reduced hepatic and muscular MDA infers that the herbal nutraceutical prevents hepatic and muscular oxidative injury and possible cell death. This observation is in agreement with previous reports in normal and diabetic animal model (22, 46). The observed *A. paniculata*-induced attenuation of MDA level is accompanied by increased GSH and SOD, which are non-enzymatic and enzymatic antioxidants, respectively. This shows that *A. paniculata* possibly dampens oxidative stress by enhancing cellular antioxidant, thus promoting cellular stability. It is plausible to infer that the ability of *A. paniculata* to maintain redox balance and confer protection on hepatic and muscular tissues accounts for the improved hepatic and muscular integrity noted in this study evidenced by improved hepatic and muscular injury markers.

Increasing pieces of evidence has shown that oxidative stress could be a cause and/or a consequence of inflammation (34, 47, 48). One way by which oxidative stress elicits inflammation is via activation of redox-sensitive transcription factor such as nuclear factor kappa-light-chain enhancer of activated B cells (NF-kB). Oxidative stress triggers the translocation of NF-kB into the nucleus and induces the transcription of several deleterious genes (49), thus NF-kB acts as a key mediator of inflammatory responses (50). Interestingly, prototypical activators of NF-kB in the classical pathway include pro-inflammatory mediators such as TNF-α and IL-1β (51–53). Activation of NF-kB may also induce pro-oxidant genes (54). Hence, the decrease in TNF-α and IL-1β in the present study is also noteworthy. This may imply that *A. paniculata* administration inactivated NF-kB signaling either by preventing oxidative stress and pro-inflammatory response. It is also likely that *A. paniculata* suppresses NF-kB signaling by reducing LDL-C levels. Oxidized LDL (OxLDL) may activate NF-kB via binding with lipooxygenase-1 (LOX-1) (4, 55).

Astonishingly, our findings that *A. paniculata* improves cellular redox state and inflammatory responses are associated with increased circulating NO. This observation is pertinent. It has been reported that high levels of HDL-C improves endothelial function by enhancing NO biosynthesis and bioavailability by downregulating free radical generation, hence preventing the negative effect of LDL-C in the vasculature (40, 56). NO is a known vasodilator (57) that improves blood flow and tissue perfusion, thus enhancing cellular glucose uptake. Therefore, it is safe to imply that *A. paniculata* improved glucose regulation, at least partly, by improving cellular glucose uptake via NO-dependent increase in tissue perfusion.

The most striking findings in this study are that *A. paniculata* treatment upregulated the activities of carbohydrate-metabolizing enzymes as well as GLUT 4 protein expression. This seems to be the first study, to the best of our knowledge that demonstrates the effect of *A. paniculata* on carbohydrate-metabolizing enzymes, particularly phosphofructokinase (the rate limiting enzyme in glycolysis), and GLUT 4 protein expression. These findings reveal that *A. paniculata* exerts a positive influence on glucose metabolism and utilization. Insulin-induced GLUT 4 translocation and glucose uptake via insulin receptor substrate (IRS) may be impaired by the activation of serine/threonine kinase cascade that triggers serine phosphorylation of IRS, which in turn impairs tyrosine phosphorylation, resulting in IR via phosphatidylinositol 3-kinase (PI3K)/Akt signaling (6). It is thus possible that *A. paniculata* downregulated PI3K/Akt signaling, with resultant enhanced insulin sensitivity and upregulated GLUT 4 protein expression. Taken together, the glucoregulatory potential of *A. paniculata* involves multiple pathways, resulting in improved glucose metabolism.

The current findings that the *A. paniculata* improves glycemic control and cardiovascular health is convincing, especially in the context of managing metabolic syndrome and T2DM. More so, the fact that body weight remained unchanged despite improvements in glucose and lipid profiles suggests that these effects are independent of weight loss. The relationship between gluco-lipid regulation and weight gain is complex. Impaired glucose tolerance may lead to increased circulatory insulin levels in an attempt to ensure optimal gluco-lipid regulation (58). The chronic hyperinsulinemia in such case facilitates fat storage, leading to weight gain (59). On the other hand, optimal insulin sensitivity inhibits weight gain. In this study, it was observed that *A. paniculata* stimulated β cell evidenced by increased β cell function and insulin levels. *A. paniculata* also enhanced insulin sensitivity and upregulated the activities of enzymes involved in glucose metabolism. Thus, the unaltered weight following *A. paniculata* administration may be due to its ability to increase insulin secretion as well as insulin sensitivity, leading to the inhibition of fat storage and weight gain.

The glucose-lowering effect of *A. paniculata* via enhancement of insulin sensitivity and GLUT 4 expression may be ascribed to its constituent phytochemicals such as dipertenoids, flavonoid, alkaloid, and tannins. Studies have established that Andrographolide is the primary active component and the major dipertenoid found in *A. paniculata*. It has been shown to exert hypoglycaemic effect via modulating Akt-NF-kB-dependent oxido-inflammation (60). Zhang et al. (61) also demonstrated that *A. paniculata* prevented incident diabetes by maintaining T helper cells Th1/Th2/Th17 homeostasis. In addition, phenols and flavonoids have been shown to confer cellular protection and stability by improving cellular redox state (23, 62). Thus, the present findings provide an extension on the mechanistic action of flavonoid/polyphenol-rich Andrographolide-containing *A. paniculata*.

Although this study present convincing evidence demonstrating the benefits of *A. paniculata* in improving insulin sensitivity and maintaining optimal glucose level, it is limited by the absence of quantitative data on the phytochemical constituents of *A. paniculata* since it is possible that there is a variability in the quantity of the active compounds of *A. paniculata*, and the andrographolide content may also fluctuate depending on the source and method of extraction of *A. paniculata*.

**Figure 7:**
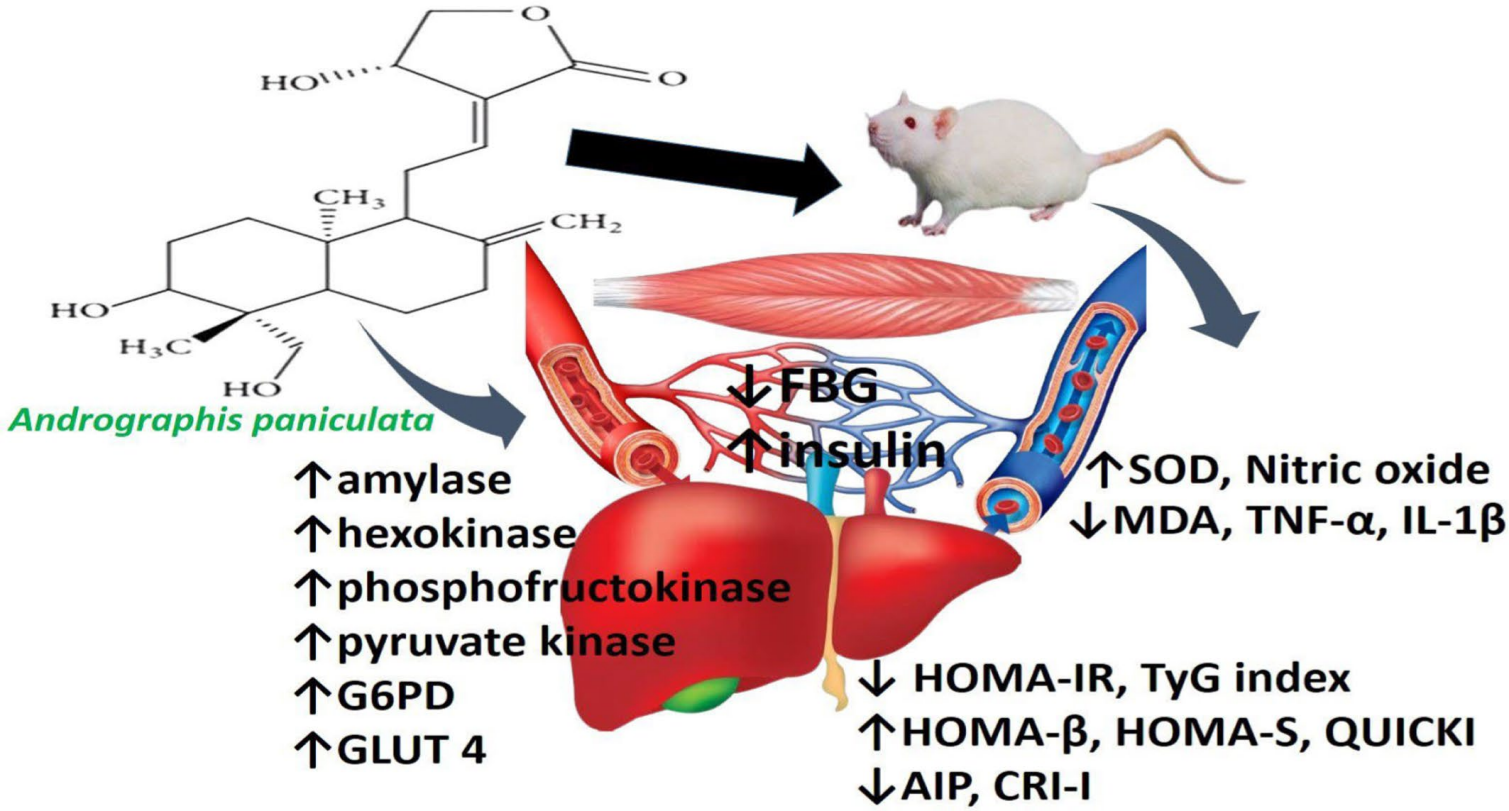
Schematic illustration of the effect of *Andrographis paniculata* on hepatic and skeletal handling of glucose and lipid.

In conclusion, the findings of this study clearly demonstrate that *A. paniculata* improves insulin sensitivity and it is associated with enhanced pancreatic β-cell function, improved atherogenic dyslipidaemia via an oxidative-sensitive signaling, which is coupled with upregulation of glycolytic enzymes, GLUT 4, and circulating NO in a non-diabetic state (Figure 7). These findings suggest that *A. paniculata* may be beneficial in maintaining optimal gluco-lipid state, thus preventing insulin resistance and incident diabetes. Also, *A. paniculata* may be explored in the management of diabetes. Experimental studies with negative and positive controls using a known anti-diabetic drug, such as metformin, as positive control may provide additional insights to the efficacy of *A. paniculata* in diabetic state. However, this may also call for caution. Monitoring fasting glycaemia during *A. paniculata* use may be important to prevent hypoglycaemia in a non-diabetic state. Although the data presented are convincing and generated from a good sample size considering that it is an experimental study using a rat model. However, generalizability of the findings to humans should be made with caution. Therefore, clinical trials are recommended to validate these findings in humans.

## Data Availability

The original contributions presented in the study are included in the article/supplementary material, further inquiries can be directed to the corresponding author.
